# Computational Analysis of GAT1 Mutations: Functional Consequences from Molecular Dynamics and Binding Free Energy Calculations

**DOI:** 10.3390/ijms262311339

**Published:** 2025-11-24

**Authors:** Muhammad Yasir, Jinyoung Park, Eun-Taek Han, Won Sun Park, Jin-Hee Han, Jongseon Choe, Mubashir Hassan, Andrzej Kloczkowski, Wanjoo Chun

**Affiliations:** 1 Department of Pharmacology, Kangwon National University School of Medicine, Chuncheon 24341, Republic of Korea; yasir.khokhar1999@gmail.com (M.Y.); jinyoung0326@kangwon.ac.kr (J.P.); 2 Department of Medical Environmental Biology and Tropical Medicine, Kangwon National University School of Medicine, Chuncheon 24341, Republic of Korea; ethan@kangwon.ac.kr (E.-T.H.); han.han@kangwon.ac.kr (J.-H.H.); 3 Department of Physiology, Kangwon National University School of Medicine, Chuncheon 24341, Republic of Korea; parkws@kangwon.ac.kr; 4 Department of Microbiology and Immunology, Kangwon National University School of Medicine, Chuncheon 24341, Republic of Korea; jchoe@kangwon.ac.kr; 5 The Steve and Cindy Rasmussen Institute for Genomic Medicine at Nationwide Children’s Hospital, Columbus, OH 43205, USA; mubashirhassan_gcul@yahoo.com (M.H.); andrzej.kloczkowski@nationwidechildrens.org (A.K.); 6 Department of Pediatrics, The Ohio State University, Columbus, OH 43205, USA; 7 Department of Biomedical Informatics, The Ohio State University, Columbus, OH 43210, USA

**Keywords:** GAT1, GAT1 mutations, tiagabine, molecular dynamics simulation, gmx_MMPBSA, epilepsy

## Abstract

GABA transporter 1 (GAT1), encoded by the *SLC6A1* gene, is essential for maintaining inhibitory neurotransmission by mediating the reuptake of GABA from the synaptic cleft. Dysfunction of GAT1 has been linked to several neurological and neurodevelopmental disorders, including epilepsy and Alzheimer’s disease. In this study, we performed a comprehensive computational investigation of reported GAT1 mutations to understand their structural and functional implications. Seven mutations (G63S, Y140C, Q291Δ, F294Δ, N310I, D451G, and G457H) were analyzed using homology modeling, structural validation tools, molecular dynamics (MD) simulation triplicates, and binding free energy calculations via the gmx_MMPBSA method. The wild-type consistently exhibited the most favorable interaction energy (−59.89 kcal/mol), the strongest binding free energy (ΔG = −28.29 kcal/mol), and the most stable hydrogen-bonding network. While all mutants displayed elevated RMSD and energy fluctuations relative to the wild-type, these changes predominantly reflected local conformational disturbances rather than global unfolding, indicating that the overall structural framework of GAT1 remains largely preserved. Among the mutants, G63S exerted the mildest effect on ligand stabilization, whereas Y140C, G457H, Q291Δ, and D451G produced substantial reductions in protein–ligand stability, weakened hydrogen bonding, and increased ligand mobility within the binding pocket. Free-energy analysis further highlighted the pronounced destabilizing influence of N310I, Q291Δ, and G457H on tiagabine binding. These findings provide mechanistic insights into how specific GAT1 mutations may alter transporter stability and function, offering a structural framework for future studies on GABAergic dysfunction and therapeutic development.

## 1. Introduction

GABA (gamma-aminobutyric acid) is the principal inhibitory neurotransmitter in the mature mammalian central nervous system. Its primary function involves reducing neuronal excitability by diminishing the likelihood of triggering an action potential. This inhibitory effect is achieved through interactions with both ionotropic GABA_A_ receptors and metabotropic GABA_B_ receptors. GABAergic neurotransmission plays a crucial role in maintaining the balance of neuronal activity within the central nervous system, contributing to the regulation of synaptic transmission and overall network stability [1,2]. The controlled release of GABA (gamma-aminobutyric acid) into the synaptic cleft is meticulously regulated to prevent overexposure and unintended diffusion to neighboring synapses. GAT1, encoded by the SLC6A1 gene, plays a crucial role in this process by facilitating the clearance of GABA from the synaptic cleft. This action, commonly known as ‘reuptake,’ involves the retrieval of GABA molecules from the synaptic cleft, ensuring the precise modulation of neurotransmitter levels and maintaining the integrity of synaptic signaling [3]. GAT1 belongs to the solute carrier 6 (SLC6) transporter family, also recognized as secondary active neurotransmitter/sodium symporters (NSSs). Other members of the NSS family, including serotonin (SERT), dopamine (DAT), glycine (GlyT) transporters, and GAT1, exhibit a relatively modest sequence identity of approximately 50%. Members of the NSS family share a common structural fold consisting of 12 transmembrane domains (TM1–12). These transporters utilize the sodium (Na^+^) and chloride (Cl^−^) gradient across the synaptic plasma membrane to facilitate their function. In this process, each substrate molecule is co-transported with Na^+^ and Cl^−^ ions, although the precise substrate-to-ion stoichiometry varies among different transporters within the family [4,5,6]. The transport mechanism of NSS (neurotransmitter/sodium symporter) follows an alternating-access mechanism. Initially, the transporter adopts an outward-open conformation, exposing the substrate-binding site to the synaptic cleft. In this state, the transporter facilitates the binding of ions and substrate molecules. This outward-open conformation allows for the selective recognition and binding of neurotransmitter substrates. The alternating-access mechanism is a dynamic process where the transporter undergoes conformational changes to transition between outward-open and inward-open states, facilitating the translocation of substrates across the synaptic plasma membrane. This mechanism ensures the precise coordination of ion and substrate binding, contributing to the regulated and controlled transport of neurotransmitters such as GABA by NSS family members like GAT1 [7]. Consequently, this process induces the closure of the extracellular gate, forming an occluded conformation. Subsequently, the intracellular gate opens, leading to the release of the substrate into the cytoplasm in an inward-open conformation.

GAT1 functions as the primary GABA reuptake transporter in the mammalian brain, with predominant expression in the cerebral cortex. It is localized in both axonal termini of neurons and astrocytes. Additionally, GAT1 is found in other neuronal tissues, including the basal ganglia [8,9]. Disturbances in the equilibrium of GABA levels within the synaptic cleft can contribute to the development of serious neurological disorders. Conditions marked by imbalances in GABA, including epilepsy, schizophrenia, anxiety, alcoholism, and depression, underscore the pivotal role of GABAergic neurotransmission in maintaining normal neuronal function and mental well-being [10,11,12].

The malfunctioning of GAT1 has been associated with neurodegenerative diseases, specifically Parkinson’s disease and Alzheimer’s disease [9,13]. Recent genetic and functional studies have revealed that mutations in GAT1 can profoundly alter transporter expression, substrate affinity, and conformational dynamics, leading to diverse neurological phenotypes. Several disease-associated missense and deletion mutations have been identified, including G63S, Y140C, Q291Δ, F294Δ, N310I, D451G, and G457H, each exerting distinct effects on GAT1 function and stability. Experimental data from radiolabeled GABA uptake assays provide critical insights into the consequences of these variants.

The G63S mutant, located within transmembrane helix 1, shows markedly reduced GABA uptake and diminished surface expression in HEK293 cells, as demonstrated by immunofluorescence and biochemical trafficking assays. These findings indicate impaired transporter trafficking to the plasma membrane and partial retention in the endoplasmic reticulum, suggesting a protein-folding defect [14,15,16,17]. The Y140C variant, positioned near the chloride-binding site in TM3, displays decreased transport turnover and altered chloride dependence in electrophysiological studies, consistent with impaired ion coordination and substrate translocation efficiency [18]. Deletions Q291Δ and F294Δ in transmembrane domain 6 have been shown through uptake and substrate-binding assays to nearly abolish GABA transport activity, with surface biotinylation analyses confirming reduced membrane expression. Structural modeling and functional assays led the original authors to conclude that these deletions destabilize the outward-open conformation, preventing proper substrate translocation [18,19]. Similarly, the N310I substitution, located near the sodium-binding site in TM6, exhibits decreased Na^+^-coupled GABA transport in Xenopus oocyte and cell-based electrophysiology assays, reflecting compromised ion coupling efficiency and reduced substrate affinity [16].

Mutations D451G and G457H, both positioned in transmembrane helix 10, have been associated with intracellular accumulation and defective folding, as confirmed by confocal imaging and protein stability assays. Functional readouts demonstrated almost complete loss of GABA uptake, leading the authors to propose that these variants disrupt helix packing required for the inward-to-outward conformational transition [18,20,21]. Collectively, these experimental observations highlight that GAT1 mutations impair either membrane trafficking, ion coupling, or conformational cycling, all of which are essential for effective GABA reuptake.

Despite these functional insights, the detailed molecular mechanisms by which these mutations perturb GAT1 structure and dynamics remain unclear. Most prior conclusions were drawn from static homology models or limited electrophysiological datasets. To address this knowledge gap, the present study applies Molecular Dynamics (MD) simulations and Molecular Mechanics Poisson–Boltzmann Surface Area (MMPBSA) calculations to elucidate the structural perturbations, conformational flexibility, and binding energetics underlying the dysfunction of these seven GAT1 mutants. This integrative computational approach provides a structural framework for understanding how pathogenic variants reshape GAT1 dynamics and disrupt GABA reuptake at the molecular level.

## 2. Results and Discussion

### 2.1. Prediction of Mutated GAT1 Structures and Structural Evaluation

**Figure 1:**
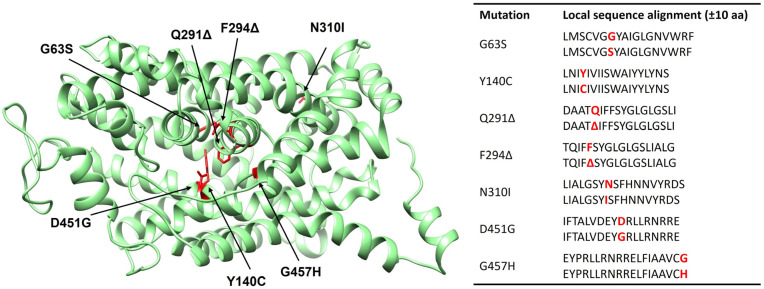
The left panel displays the three-dimensional structure of the wild-type and the location of all seven mutants (G63S, Y140C, Q291Δ, F294Δ, N310I, D451G, and G457H), indicated by a red color. The right figure displays the local sequence alignments (±10 amino acids) around each mutation, highlighting the substituted or deleted residues in red.

The amino acid sequence of human GABA transporter 1 (GAT1) was retrieved from the UniProt database (UniProt ID: P30531). Based on the available Cryo-EM structure of human GAT1 (PDB ID: 7Y7Y), seven disease-associated mutants were generated through homology modeling by substituting or deleting specific residues within the wild-type sequence. Specifically, glycine residues at positions 63 and 457 were replaced by serine (G63S) and histidine (G457H), respectively; tyrosine (Y140) was mutated to cysteine (Y140C); asparagine (N310) was substituted with isoleucine (N310I); and aspartic acid (D451) was mutated to glycine (D451G). In addition, two deletion variants were modeled at positions 291 (Q291Δ) and 294 (F294Δ) (Figure 1). These mutations were selected based on prior experimental evidence linking them to defective GAT1 expression, trafficking, or transporter function.

Since homology modeling can introduce stereochemical inconsistencies, it was essential to evaluate the accuracy and stability of the predicted mutated models before further analysis. Therefore, the modeled structures were validated using a combination of MolProbity, ProSA, and ERRAT servers, each providing complementary insights into model quality. MolProbity assesses stereochemical parameters such as bond lengths, angles, and steric clashes, providing an overall score that reflects model geometry. ProSA evaluates the energy profile of the 3D structure and compares it to experimentally determined protein structures, with its **Z-score** serving as a global measure of structural plausibility. ERRAT, on the other hand, analyzes non-bonded atomic interactions, identifying regions that deviate from ideal packing.

All seven mutant models displayed acceptable MolProbity (0.65–1.75) and ERRAT scores (~97–98%), confirming high-quality stereochemistry and favorable atomic packing. The ProSA Z-scores (−4.99 to −5.18) and Ramachandran values (97.0% to 97.9%) were comparable to values observed for native membrane proteins, suggesting that the modeled mutants maintain correct global folding (Supplementary data Figures S1 and S2). Interestingly, most mutants exhibited slightly improved ERRAT and MolProbity scores compared to the wild-type structure (Table 1), reflecting successful local optimization following residue substitution. These subtle score variations suggest that, while the overall tertiary structure remains conserved, specific mutations may induce localized distortions in the backbone geometry or residue packing, which could influence substrate recognition and transporter flexibility.

### 2.2. Functional and Stability Prediction of GAT1 Mutations

To evaluate the potential functional and structural consequences of the seven GAT1 mutations, a combination of sequence- and structure-based computational tools, PolyPhen2, MutPred2, ENCoM, DynaMut2, and DUET, was employed (Table 2). These complementary algorithms were selected to assess pathogenicity, conformational flexibility, and thermodynamic stability of each mutant model relative to the wild-type transporter.

PolyPhen2 and MutPred2, which predict the likelihood of a substitution being deleterious based on sequence conservation and physicochemical properties, classified all five substitution mutations (G63S, Y140C, N310I, D451G, and G457H) as probably damaging with scores close to 1.00. These high scores suggest strong evolutionary intolerance to amino acid changes at these positions, implying their functional importance in maintaining GAT1 transporter integrity. MutPred2 scores (0.906–0.959) further supported this, indicating a high probability that these substitutions alter molecular functions such as ion binding, transmembrane stability, or substrate interaction.

The ENCoM analysis, which estimates vibrational entropy changes (ΔS_vib), revealed differential effects of mutations on protein flexibility. Positive ΔS_vib values for G63S (0.188), N310I (0.153), and G457H (0.941) suggest a slight increase in local flexibility, potentially loosening structural rigidity near the substrate-binding or gating regions. Conversely, negative ΔS_vib values for Y140C (−0.971) and D451G (−0.457) indicate local rigidification, which could hinder conformational transitions necessary for substrate translocation. These observations align with previous experimental findings reporting reduced transport turnover and impaired folding in several of these variants.

The DynaMut2 and DUET tools were used to estimate the impact of each mutation on overall protein stability in terms of the change in Gibbs free energy (ΔΔG). Negative ΔΔG values denote destabilizing mutations, whereas positive values imply stabilization. Except for D451G, which exhibited a stabilizing effect (ΔΔG = +1.38 kcal/mol, DUET = +1.928 kcal/mol), all other substitutions showed negative ΔΔG values (−0.02 to −2.10 kcal/mol), suggesting decreased thermodynamic stability relative to the wild type. The G457H mutation, in particular, showed the strongest destabilization (−1.23 kcal/mol in DynaMut2 and −2.107 kcal/mol in DUET), which correlates with its experimentally reported misfolding and intracellular retention.

The deletion mutations (Q291Δ and F294Δ) could not be evaluated by these algorithms due to their structural complexity. However, these predictive analyses indicate that most GAT1 mutations are functionally deleterious and structurally destabilizing. The combination of high pathogenicity scores and negative stability values suggests that these mutations may perturb folding efficiency, transmembrane packing, and ion-coupled conformational transitions. These computational findings provide a molecular basis for understanding how specific amino acid alterations compromise GAT1 function, guiding further investigation through molecular dynamics simulations and binding energy analyses.

### 2.3. Binding Site Prediction of GAT1

**Figure 2:**
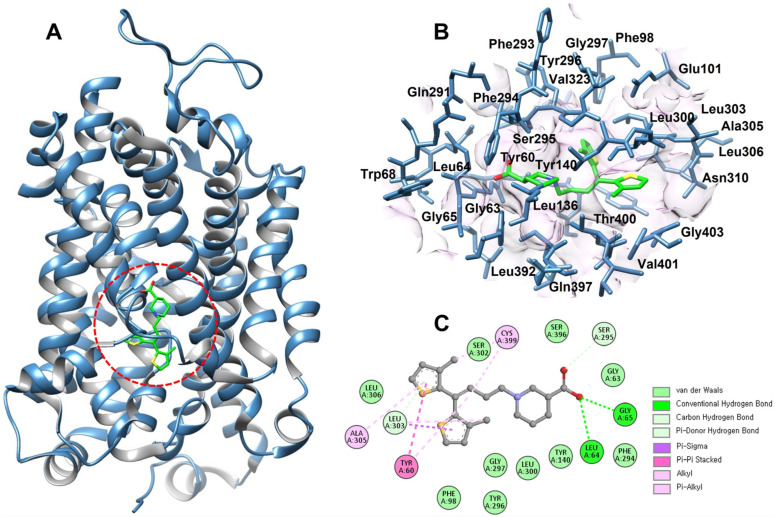
Structural representation of the tiagabine–GAT1 binding interactions. (**A**) Overall 3D architecture of the GAT1 transporter, highlighting the ligand-bound cavity (red dashed circle). (**B**) Close-up view of key residues surrounding tiagabine (green), showing the detailed binding pocket environment. (**C**) 2D interaction map illustrating hydrogen bonds, hydrophobic contacts, and π-interactions between tiagabine and essential amino acid residues that stabilize the complex.

The function of a binding pocket is dictated by the collection of amino acid residues around it, as well as its shape and placement inside a protein [22,23]. The binding pocket residues of GAT1 involved in tiagabine recognition were identified based on previously reported structural studies [15]. Key residues, including Y60, A61, G63, G65, L136, Y140, F294, S295, Y296, G297, L300, S396, and T400, were selected and validated using Discovery Studio and UCSF Chimera to ensure accurate spatial mapping of the inhibitor-binding region (Figure 2). Notably, several of the investigated mutations, G63S, Y140C, Q291Δ, F294Δ, and N310I, are located in close (within the 7Å) proximity to the tiagabine-binding pocket, suggesting their potential influence on ligand affinity and transporter inhibition.

Given that tiagabine remains the only clinically approved GAT1 inhibitor, analyzing these mutations near its binding site is particularly significant. Alterations in these residues may modify hydrogen bonding, hydrophobic packing, or local conformational dynamics within the pocket, thereby impacting drug recognition and GABA transport efficiency. This spatial correlation supports the hypothesis that disease-associated GAT1 variants may not only disrupt transporter function but could also influence the binding characteristics of therapeutic inhibitors.

### 2.4. Molecular Docking Analysis

To evaluate how individual mutations affect the interaction of tiagabine with GAT1, molecular docking was performed for the wild-type and all seven mutant models before molecular dynamics (MD) simulations. Tiagabine was docked into each structure, and the resulting complexes were analyzed based on their docking energy values (Supplementary data Table S1).

Molecular docking was performed to evaluate how each GAT1 mutation influences the initial binding compatibility of tiagabine before MD simulations. The wild-type model showed the strongest affinity, reflected by the lowest Boltz-2 score (−1.2298) and a Vina energy of −6.05 kcal/mol, confirming its optimal fit for tiagabine. Mutants such as G63S, Y140S, Q291Δ, and F294Δ showed only moderate reductions in affinity, indicating mild local alterations around the binding pocket. In contrast, N310I, D451G, and G457H showed more pronounced disruptions. N310I produced a positive Boltz-2 value, suggesting thermodynamic instability despite the strong Vina score of −6.85 kcal/mol. This discrepancy suggests that while the ligand fits well sterically within the modified pocket, local energetic penalties arising from altered polarity or hydrophobicity may reduce overall binding reliability. The D451G mutation showed a large negative shift in Boltz-2 affinity (−0.8051), reflecting a pronounced disruption of the electrostatic environment surrounding the ligand; however, its Vina score (−6.80 kcal/mol) indicates that favorable interactions can still occur transiently. G457H demonstrated weakened binding in both scoring methods, indicating that changes in electrostatics or steric properties significantly reduced ligand compatibility. Overall, the docking results highlight that while several mutations only slightly affect tiagabine binding, others, especially N310I, D451G, and G457H, substantially perturb the ligand-binding environment.

### 2.5. Structural Impact of Mutations on the GAT1–Tiagabine Complex

**Figure 3:**
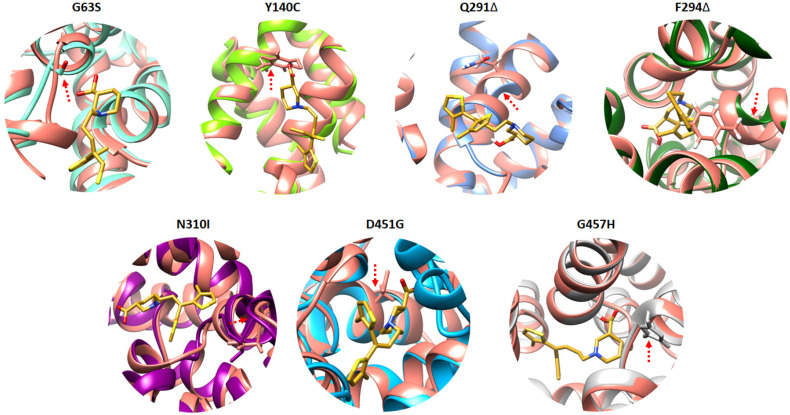
This figure displays the superimposed mutated models, where wild-type amino acid residues and mutated residues are represented by showing side chains. Additionally, the figure indicates the location of all mutated residues in the wild-type GAT1. The location is represented by the red arrow. The bound tiagabine was colored gold.

Further examination of the predicted GAT1 mutations revealed distinct structural consequences within the tiagabine-binding region, providing critical insights into how specific residue alterations may affect transporter function and drug recognition. As illustrated in Figure 3, tiagabine (shown in yellow) remained bound within the central cavity across all models, allowing a direct comparison of local conformational changes between the wild-type and mutant complexes.

Deletion mutations such as Q291Δ and F294Δ induced pronounced distortions in the backbone conformation adjacent to the binding pocket. These regions are structurally important for maintaining the integrity of the transmembrane helices that define the substrate and inhibitor cavity. The observed deformation likely disrupts the optimal alignment of key residues involved in substrate gating and ligand stabilization. Such deletions may reduce the overall flexibility or create steric voids that destabilize tiagabine binding, thereby impairing transporter efficiency or drug responsiveness. In contrast, substitution mutations (G63S, Y140C, N310I, D451G, and G457H) predominantly altered the orientation and polarity of side chains near the ligand-binding site. These modifications may reshape the local electrostatic landscape, influencing hydrogen bonding and hydrophobic interactions critical for ligand affinity. For example, substitution of small or nonpolar residues with bulkier or polar ones could weaken van der Waals contacts or introduce new hydrogen bond donors/acceptors, subtly altering tiagabine’s binding pose and energetic stability.

Taken together, these results suggest that deletion mutations primarily disrupt the structural framework of the binding cavity, while substitution mutations fine-tune the chemical environment within it. This dual impact underscores the delicate structural balance required for GAT1 functionality and inhibitor recognition. From a mechanistic standpoint, it may be hypothesized that mutations such as Q291Δ and F294Δ could impair the conformational transitions necessary for GABA uptake, while side-chain substitutions might lead to partial loss or alteration of inhibitor sensitivity. These findings collectively support the idea that even minor mutations near the tiagabine-binding site can significantly affect binding affinity, transporter stability, and possibly pharmacological efficacy, which could have implications for drug resistance or altered therapeutic outcomes in GAT1-associated disorders.

### 2.6. Molecular Dynamics (MD) Simulations

All seven mutant GAT1 models, along with the wild-type structure, were subjected to all-atom molecular dynamics (MD) simulations to evaluate their structural stability, conformational flexibility, and dynamic behavior. To ensure robustness and statistical reliability, three independent MD replicates (triplicates) were performed for each system.

#### 2.6.1. RMSD Backbone to the Backbone

**Figure 4:**
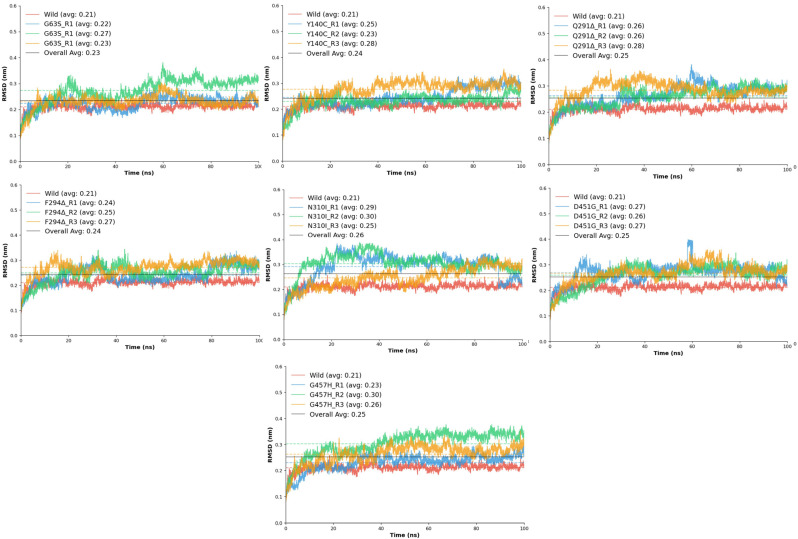
RMSD backbone trajectories of the mutated model during 100ns MD triplicates in comparison with the wild model.

The root mean square deviation (RMSD) analysis was performed to assess the structural stability and conformational flexibility of the wild-type GAT1 and its seven mutant variants (G63S, Y140C, Q291Δ, F294Δ, N310I, D451G, and G457H) throughout the molecular dynamics (MD) simulations (Figure 4). This analysis provided quantitative insight into the backbone deviations that occurred as a result of each mutation, allowing direct comparison with the native transporter to evaluate the impact of residue substitutions and deletions on overall stability.

The wild-type GAT1 displayed an average RMSD of 0.21 ± 0.01 nm, indicating a stable conformation with minimal fluctuation throughout the simulation period. This structural consistency reflects the native protein’s optimal folding and robust intramolecular interactions required for functional integrity. In contrast, all mutant systems exhibited slightly elevated RMSD values, signifying varying degrees of conformational perturbation. Among these, the N310I mutant showed the highest deviation (0.26 nm) and the lowest stability score (0.951), suggesting pronounced disruption in local packing and hydrogen bonding. The introduction of a hydrophobic isoleucine in place of asparagine likely introduced steric constraints that disturbed the local transmembrane environment. Similarly, D451G and G457H demonstrated noticeable fluctuations (average RMSD ≈ 0.25 nm), accompanied by reduced stability (0.956–0.967), consistent with increased backbone mobility near the transmembrane helices.

The deletion mutants Q291Δ and F294Δ exhibited moderate deviations (average RMSD ≈ 0.24–0.25 nm), reflecting partial destabilization of the surrounding secondary structure. These results align with the hypothesis that deletions in transmembrane domain 6 can perturb helical alignment, which may alter substrate translocation dynamics [17]. Meanwhile, G63S and Y140C displayed only mild fluctuations (average RMSD ≈ 0.23–0.24 nm) relative to the wild-type model. These substitutions likely cause localized effects without significantly disturbing the overall fold, as the introduction of a small polar residue (serine or cysteine) primarily influences nearby hydrogen bonding networks rather than the global conformation. Comparatively, all mutations exhibited slightly higher RMSD values and lower stability scores than the wild-type, emphasizing their collective impact on the conformational equilibrium of GAT1.

#### 2.6.2. Root Mean Square Fluctuation (RMSF) Analysis

The RMSF analysis was conducted to evaluate the residue-level flexibility of the wild-type and mutant GAT1 models during molecular dynamics simulations. RMSF provides insight into the local dynamic behavior of protein regions, where elevated values indicate increased atomic motion and potential loss of structural rigidity. The wild-type GAT1 exhibited a mean RMSF of 0.10 ± 0.04 nm, with fluctuations ranging between 0.05 and 0.37 nm, confirming its high conformational stability and serving as the reference for comparison.

All mutant systems demonstrated slightly elevated RMSF values relative to the wild-type model, signifying increased flexibility in specific regions of the transporter. Among these, the D451G and F294Δ mutations produced noticeable increases in local fluctuations, with mean RMSF values around 0.11–0.13 nm and maximum deviations exceeding 1.0 nm, reflecting moderate to high destabilization of backbone atoms near the mutation sites. The Q291Δ deletion mutant showed the most pronounced flexibility, with a maximum fluctuation of 1.18 nm and stability scores dropping to 0.914, suggesting that the removal of Gln291 significantly perturbs the local secondary structure and packing interactions. Similarly, the N310I mutant exhibited enhanced residue mobility (mean RMSF ≈ 0.13 nm) and reduced stability, likely due to the substitution of polar asparagine with hydrophobic isoleucine, which disrupts local hydrogen bonding and introduces steric strain within the transmembrane region.

**Figure 5:**
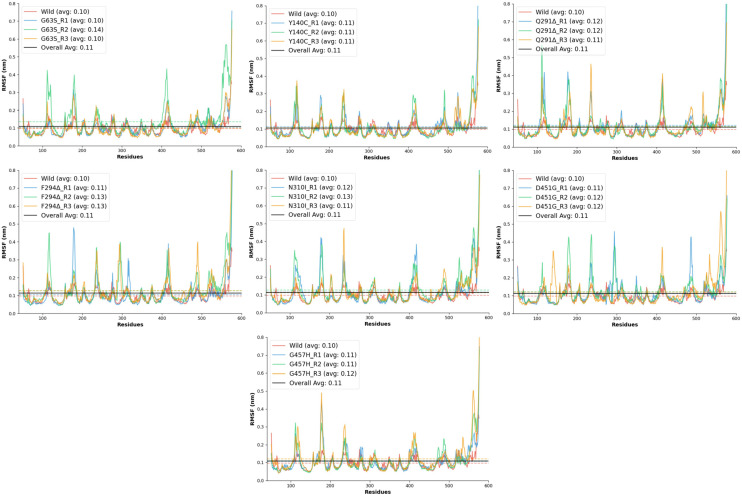
RMSF graphs of the predicted mutated structures from the N-terminal to the C-terminal residues. The average values have also been depicted in the graph legends for all three independent triplicates.

The G457H and D451G substitutions, both located in the transmembrane domain, displayed moderate increases in flexibility (maximum RMSF 0.85–1.06 nm), indicating that these mutations may induce partial relaxation of nearby helices or loops. By contrast, G63S and Y140C showed relatively conservative dynamic changes (Figure 5), maintaining mean RMSF values close to the wild-type (~0.10–0.11 nm). These results imply that substitutions involving small polar residues primarily affect local hydrogen-bonding patterns without causing significant global structural distortion.

Overall, all mutant models exhibited RMSF values slightly higher than the wild-type, confirming that these mutations collectively reduce local rigidity and increase atomic fluctuations within GAT1. Among them, Q291Δ, N310I, F294Δ, and D451G emerged as the most destabilizing variants, while G63S and Y140C remained comparatively stable.

#### 2.6.3. The Radius of Gyration (R_g_)

The R_g_ analysis was conducted to evaluate the overall compactness and conformational stability of the wild-type and mutant GAT1 models during molecular dynamics simulations. Stable R_g_ values with low fluctuations indicate well-folded protein structures and minimal large-scale conformational changes [24]. The wild-type GAT1 displayed an average R_g_ of 2.33 ± 0.01 nm, ranging between 2.31 and 2.38 nm, with a stability score of 0.990, confirming a compact and structurally consistent conformation throughout the simulation. When compared to the wild-type, all mutant systems exhibited only minor variations in R_g_, suggesting that none of the substitutions or deletions caused major unfolding or global destabilization.

Among the mutants, D451G displayed slightly higher R_g_ values (2.35–2.37 nm), implying mild structural expansion but overall retained compactness. The F294Δ deletion mutant also exhibited a modest increase in R_g_ (2.35–2.36 nm). The G63S substitution maintained R_g_ values closely aligned with the wild-type (2.35–2.36 nm) and displayed excellent stability (0.990–0.991), indicating negligible impact on global compactness. Similarly, G457H and N310I mutants demonstrated mean R_g_ values around 2.35–2.36 nm, confirming that these substitutions do not significantly perturb the tertiary structure.

The Y140C mutant showed particularly consistent behavior, maintaining a narrow R_g_ range (2.34–2.36 nm) while achieving one of the highest stability scores, indicating enhanced rigidity or structural reinforcement. The Q291Δ deletion mutant also maintained compactness with an average R_g_ of 2.34 nm and stable replicates (0.991–0.992), suggesting that deletion at this position induces negligible global structural relaxation.

**Figure 6:**
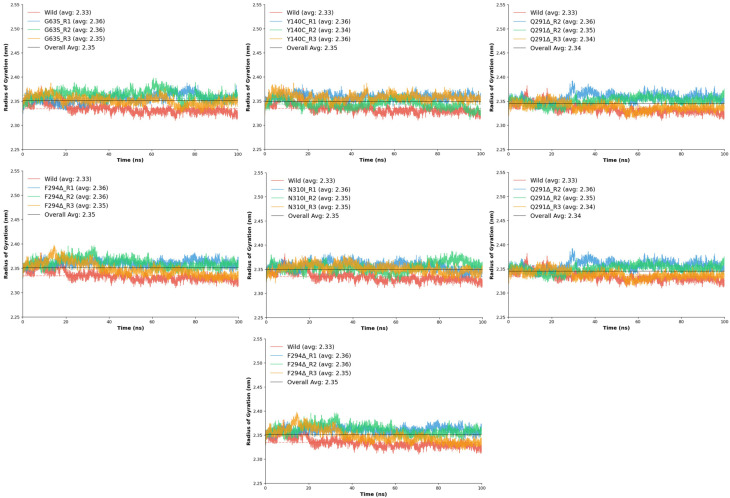
The R_g_ values G63S, Y140C, N310I, Q291Δ, F294Δ, G451G, and G457H in comparison with wild type are represented in graphical form.

Collectively, the R_g_ analysis demonstrates that all seven mutants preserve a similar degree of compactness to the wild-type (Figure 6), with mean R_g_ values clustered between 2.34 and 2.36 nm. Subtle increases in stability observed in certain mutants, particularly Y140C, suggest potential improvements in local packing or rigidity, while none of the variants exhibited conformational expansion indicative of destabilization. These findings indicate that although the mutations introduce local perturbations observed in RMSD and RMSF analyses, the global architecture of GAT1 remains structurally robust, implying that functional alterations likely arise from localized dynamic effects rather than from large-scale structural disruption.

#### 2.6.4. RMSD GAT1-Tiagabine Analysis

The RMSD analysis of the GAT1–tiagabine complexes was performed to assess the conformational stability and dynamic integrity of ligand binding throughout molecular dynamics simulations. This analysis provided insight into how specific amino acid substitutions and deletions affect tiagabine accommodation within the transporter binding pocket compared to the wild-type complex. The wild-type GAT1–tiagabine complex exhibited an average RMSD of 0.19 ± 0.02 nm, with a maximum deviation of 0.27 nm, reflecting minimal positional drift of the ligand and confirming strong, persistent binding throughout the simulation. This model served as the baseline for evaluating the influence of each mutation on ligand stabilization.

Among the mutants, all exhibited higher RMSD values than the wild-type, suggesting that the mutations introduced varying degrees of ligand destabilization. The D451G variant displayed a marked increase in deviation, with average RMSD values between 0.35 ± 0.08 nm and 0.42 ± 0.07 nm, indicating weakened ligand retention. The loss of the negatively charged aspartate residue likely disrupted electrostatic anchoring and hydrogen bonding with tiagabine, reducing its overall stabilization. The F294Δ deletion mutant exhibited moderate deviations (0.28 ± 0.07–0.34 ± 0.03 nm) and stability values of 0.933–0.966, suggesting that removal of the bulky phenylalanine side chain slightly affected hydrophobic interactions but did not fully destabilize the complex.

**Figure 7:**
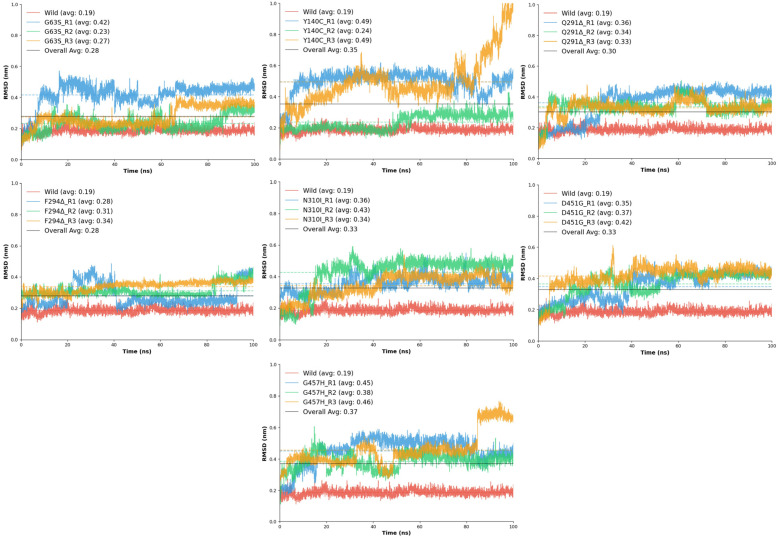
The RMSD plots of the tiagabine bound to the GAT1 backbone. The graphs display the three independent replicates (R1–R3) for a given mutant alongside the wild-type reference for the comparison.

The G63S mutation demonstrated RMSD values ranging from 0.23 ± 0.05 to 0.42 ± 0.07 nm, indicating modest perturbations, suggesting that the mutation’s effect is localized and does not strongly impact global binding stability. In contrast, the G457H mutant exhibited the largest RMSD fluctuations, averaging 0.38–0.46 nm (Figure 7, with stability scores dropping to 0.904–0.950. The bulkier histidine residue likely caused steric interference and altered local electrostatics, leading to substantial ligand displacement events, as reflected by the high RMSD observed in one replicate (0.77 nm).

Similarly, the N310I substitution produced elevated RMSD values (0.34–0.43 nm) and lower stability (0.906–0.952), indicating reduced affinity due to the loss of polar contacts and changes in side-chain hydrophobicity. The Q291Δ deletion displayed comparable instability, with RMSD averages around 0.30 nm and stability values between 0.905 and 0.950, implying that loss of Gln291 may cause subtle backbone rearrangements that affect tiagabine positioning within the binding site. The Y140C mutant exhibited the greatest range of fluctuation, with RMSD values spanning 0.24–0.49 nm, indicating severe dynamic instability. The replacement of the aromatic tyrosine with a smaller cysteine residue weakened π–π stacking and hydrophobic contacts critical for ligand anchoring, resulting in transient ligand displacements during the simulation.

When compared collectively, the wild-type complex retained the highest stability and lowest RMSD, confirming optimal structural compatibility with tiagabine. Mutants such as G63S and F294Δ showed relatively mild deviations, suggesting minimal disruption of ligand-binding geometry, whereas G457H, Y140C, and N310I demonstrated pronounced structural fluctuations, highlighting their significant impact on binding pocket integrity. Overall, the RMSD trends indicate that mutations altering electrostatic, aromatic, or hydrophobic residues within the tiagabine-binding region compromise the structural fidelity of the GAT1–inhibitor complex. These results reinforce that the wild-type conformation provides the most stable environment for tiagabine binding, while specific substitutions, particularly G457H, Y140C, and N310I, destabilize ligand interactions and may impair inhibitor efficacy in mutated GAT1 variants.

#### 2.6.5. Hydrogen Bond Analysis

The hydrogen-bond analysis of the GAT1–tiagabine complexes provided additional mechanistic insight into how each mutation alters ligand stabilization during the simulation. Hydrogen bonds were evaluated both as fully satisfied interactions (Actual hydrogen Bonds), meeting strict geometric criteria, and as potential interactions (Potential Hydrogen Bonds), where donor–acceptor distances fall within a favorable threshold but angular requirements are not met. In the wild-type complex, tiagabine maintained the most consistent pattern of both actual and potential hydrogen bonds, confirming a stable and persistent interaction environment that supports strong ligand retention and complements the energetic and structural analyses.

Across the mutants, hydrogen-bonding behavior was markedly altered, reflecting varying degrees of destabilization within the GAT1–tiagabine complex. G63S showed intermittent actual H-bonds and numerous potential contacts, indicating fluctuating ligand alignment due to subtle local rearrangements. Y140C displayed an even stronger reduction in actual hydrogen bonding, highlighting the crucial role of Tyr140 in maintaining proper ligand orientation. The Q291Δ and F294Δ deletions both caused substantial loss of stable interactions; although transient contacts occurred—particularly in F294Δ between 20 and 50 ns—they rarely matured into persistent H-bonds, reflecting increased local flexibility near the binding site. N310I produced many potential but few actual interactions, suggesting that steric and polarity changes disrupted proper geometric alignment. D451G showed the most severe decline, with almost no actual H-bonds and minimal potential contacts, indicating major electrostatic disruption. G457H exhibited frequent potential but limited stable bonds, implying that the histidine substitution hindered consistent ligand positioning despite maintained proximity to the binding pocket (Supplementary Figure S3).

**Figure 8:**
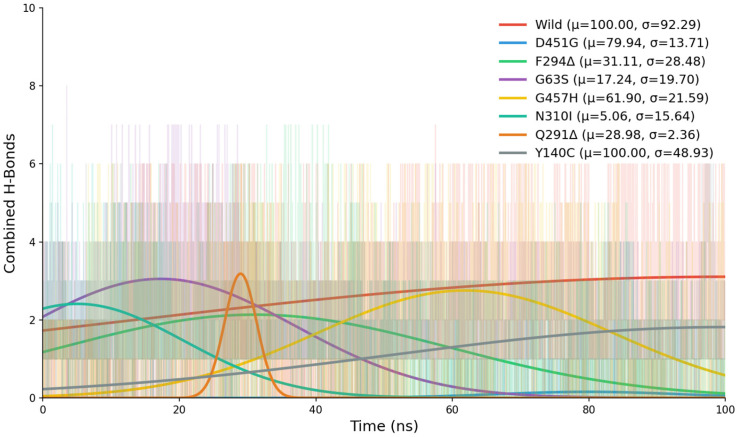
Time-resolved hydrogen-bonding analysis of tiagabine across wild-type and mutant GAT1 systems.

Moreover, the Gaussian analysis revealed distinct temporal and stability patterns in hydrogen bonding. The wild-type model showed no clear peak (μ = 100 ns) and a broad distribution (σ = 92.29), indicating fluctuating yet sustained hydrogen bonding throughout the simulation. The D451G and G457H mutants exhibited later or mid-simulation peaks (μ ≈ 80 ns and 62 ns, respectively) with moderate to low σ values, reflecting comparatively localized and more stable bonding episodes. In contrast, F294Δ, G63S, and N310I displayed early peaks (μ = 31 ns, 17 ns, and 5 ns, respectively) with varying degrees of instability; moderate σ in F294Δ and G63S suggested early but fluctuating rearrangements, while the lower σ in N310I pointed to a brief, consistent hydrogen-bonding phase that rapidly diminished. Q291Δ showed the sharpest early peak (μ = 29 ns) and extremely low σ (2.36), indicating a highly localized but short-lived stable interaction. Y140C resembled the wild-type pattern (μ = 100 ns) but with a moderately broad σ (48.93), reflecting persistently unstable hydrogen bonding across the trajectory. Collectively, these profiles demonstrate how each mutation distinctly alters the timing, persistence, and stability of hydrogen-bond formation within the GAT1–tiagabine complex (Figure 8).

Overall, these results reveal a clear relationship between hydrogen-bonding behavior and the structural or energetic stability of the GAT1–tiagabine complexes.

#### 2.6.6. MD Interaction Energy Analysis

**Figure 9:**
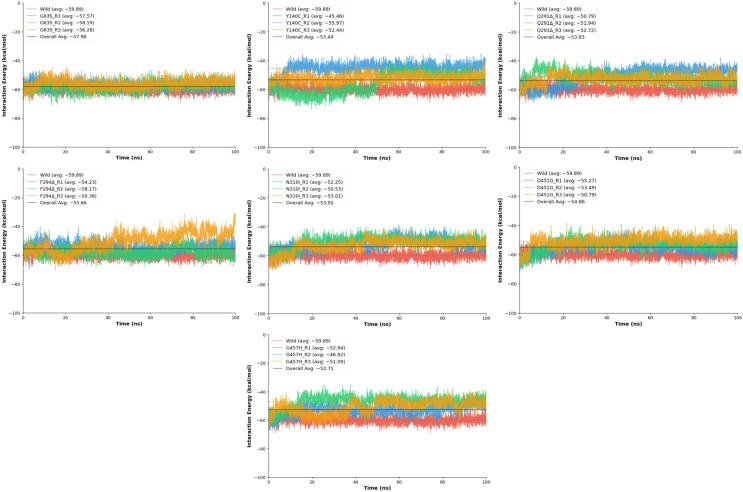
MD interaction profile of the mutated models. Each panel displays the three independent replicates (R1–R3) for a given mutant alongside the wild-type reference.

The protein–ligand interaction energy analysis was performed to determine how point and deletion mutations affect the binding affinity and dynamic stability of Tiagabine within the GAT1 binding pocket during molecular dynamics simulations. Interaction energies were computed for the wild-type and seven mutant models (D451G, F294Δ, G63S, G457H, N310I, Y140C, and Q291Δ), where more negative energy values represent stronger ligand binding over time (Figure 9). The wild-type GAT1–Tiagabine complex exhibited the strongest and most stable binding profile, with a mean interaction energy of −59.89 ± 2.72 kcal/mol, confirming a robust and well-maintained ligand association throughout the simulation. Compared to this baseline, most mutants demonstrated reduced binding affinity and greater energetic fluctuation, suggesting that specific amino acid substitutions and deletions compromise the stability of the ligand-binding pocket.

Among the mutants, G63S showed the most conserved interaction profile, with average energies ranging from −56.28 to −58.19 kcal/mol and stability scores between 0.232 and 0.254, nearly matching the wild-type performance. This indicates that the replacement of glycine with serine causes only minimal perturbation, preserving the native hydrogen-bonding environment and Tiagabine positioning. Conversely, several other mutations led to noticeable destabilization. The D451G and N310I variants both exhibited moderate decreases in interaction energy (−50 to −53 kcal/mol) and reduced stability scores (≈0.19), likely due to the loss of polar contacts and impaired electrostatic complementarity within the binding pocket. These substitutions appear to disrupt key side-chain interactions that contribute to ligand recognition. More pronounced effects were observed in the F294Δ, Q291Δ, Y140C, and G457H mutants. The F294Δ deletion resulted in fluctuating interaction energies (−50.36 to −58.17 kcal/mol) and variable stability, with one replicate displaying considerable energetic instability. Similarly, Q291Δ exhibited large energy variations and low stability (as low as 0.142), implying that deletion of Gln291 alters local backbone geometry and reduces ligand accommodation efficiency. The Y140C mutation produced the highest variability in interaction energy, ranging from −45.46 to −55.97 kcal/mol, with notable instability due to the loss of π–π and hydrogen bonding interactions that tyrosine typically provides in ligand anchoring. Similarly, G457H also demonstrated a substantial reduction in binding affinity and increased conformational fluctuation.

### 2.7. gmx_MMPBSA Binding Free Energy Analysis

The binding free energy (ΔG) analysis was conducted to quantitatively assess the strength and stability of tiagabine interactions within the wild-type and mutant GAT1 complexes. The computed ΔG values, derived from molecular dynamics trajectories, provide a thermodynamic measure of ligand affinity, where more negative values indicate stronger and more favorable binding interactions. Consistent with the structural and energetic analyses, the wild-type GAT1–tiagabine complex exhibited the most favorable free energy of binding, averaging −28.29 kcal/mol, confirming a highly stable and energetically optimized interaction between tiagabine and the native transporter configuration.

Across the mutant models, varying degrees of reduction in binding free energy were observed, indicating that each mutation influenced ligand affinity to a different extent. The G63S variant showed the least deviation from the wild-type, with an average ΔG of −26.86 kcal/mol, suggesting that substitution of glycine with serine has only a minor effect on binding energetics, consistent with earlier findings of limited structural disruption. Similarly, the F294Δ deletion mutant maintained moderate affinity (−25.70 kcal/mol), implying that loss of the phenylalanine residue slightly weakened hydrophobic interactions but did not severely compromise overall binding stability. The Y140C mutation also produced comparable binding energy (−25.33 kcal/mol) (Table 3), reflecting moderate destabilization likely due to loss of aromatic stacking and hydrogen bonding contributions from the tyrosine side chain.

**Figure 10:**
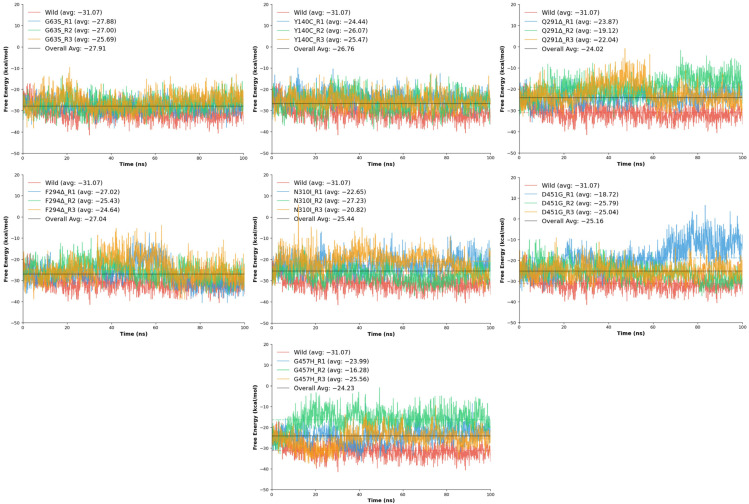
Free energy interaction profiles of tiagabine with the wild-type GAT1 and seven mutants over 100 ns MD simulations. Each panel displays the three independent replicates (R1–R3) for a given mutant alongside the wild-type reference, with average interaction energies indicated in the legends. The plots illustrate mutation-induced variations in binding stability relative to the wild-type transporter.

In contrast, several mutants exhibited substantially less favorable ΔG values, indicating weakened ligand binding (Figure 10). The N310I mutation showed an average ΔG of −23.57 kcal/mol, highlighting reduced interaction energy resulting from the loss of polar contacts and the introduction of a hydrophobic residue within the binding pocket. Similarly, D451G displayed a mean ΔG of −23.18 kcal/mol, consistent with disruption of electrostatic stabilization arising from the loss of the negatively charged aspartate side chain. The Q291Δ deletion and G457H substitution showed the lowest binding affinities among all variants, with average ΔG values of −21.68 kcal/mol and −21.94 kcal/mol, respectively. Both mutations likely induce local conformational rearrangements that misalign critical residues within the binding site, diminishing favorable interactions with tiagabine and reducing overall complex stability.

Overall, the free energy results reinforce the trend observed in the RMSD and interaction energy analyses, where the wild-type complex maintains the most energetically favorable and stable binding conformation. Mutations such as G63S, Y140C, and F294Δ exert moderate effects on tiagabine affinity, while Q291Δ, N310I, D451G, and G457H significantly weaken ligand binding, reflecting the sensitivity of the GAT1 binding pocket to both side-chain polarity and structural integrity. Collectively, these thermodynamic outcomes suggest that even subtle mutations within or near the tiagabine-binding cavity can meaningfully alter the energetic landscape of inhibitor binding, potentially impacting the transporter’s pharmacological responsiveness.

## 3. Methods

### 3.1. Structure Prediction of Human GABA Transporter 1

GABA transporter 1 (GAT1), alternatively referred to as sodium- and chloride-dependent GABA transporter 1, is a protein encoded by the SLC6A1 gene in humans, comprises 599 amino acid residues. The amino acid sequence of GAT1 was accessed from uniport and the 3D structure prediction was carried through SWISS-MODEL. The electron microscopic structure of human GAT1 (PDB-7Y7Y) was selected to be a reference structure for 3D model prediction of wild and mutated structures.

All the selected mutated models (G63S, Y140C, Q291Δ, F294Δ, N310I, D451G, and G457H) and the wild structures were built and further minimized by Discovery Studio and UCSF Chimera. The stereochemical properties of the predicted mutated structures were rigorously evaluated using the MolProbity server (http://molprobity.biochem.duke.edu/index.php) (accessed on 26 December 2023) [25] and ProSA-web (https://prosa.services.came.sbg.ac.at/prosa.php) (accessed on 8 January 2024) [26]. The MolProbity server provided essential information, including Ramachandran plots and values, shedding light on the conformational quality of the structures. Additionally, ProSA-web facilitated the assessment of overall model quality and potential errors.

### 3.2. Stability Changes Prediction of Mutated Models

To evaluate the structural and functional impact of the selected mutations on GAT1, a panel of complementary bioinformatic tools was employed, each providing distinct predictive capabilities. PolyPhen-2 [27] was used to assess the potentially damaging effects of amino acid substitutions based on sequence conservation, structural features, and comparative evolutionary profiles. This tool integrates multiple physical and comparative parameters to classify mutations as benign, possibly damaging, or probably damaging. MutPred2 [28], a machine-learning–based predictor trained on pathogenic and neutral human variants, was utilized to estimate the pathogenic potential of each substitution. In addition to providing a general probability score, MutPred2 incorporates an elastic network model to estimate vibrational entropy changes, offering insight into mutation-induced alterations in protein dynamics and stability.

To further examine the thermodynamic effects of the mutations, DynaMut2 [29] was applied. This updated version of DynaMut combines normal mode analysis with statistical potentials to predict changes in folding free energy (ΔΔG) and alterations in protein flexibility arising from point mutations. DUET [30], a consensus method integrating two independent stability predictors, mCSM and SDM, was also employed to obtain robust estimations of ΔΔG values by leveraging structural environment parameters and evolutionary conservation simultaneously. The integration of these predictors provided a comprehensive stability profile across diverse computational frameworks.

Finally, the structural context and spatial distribution of each variant were visualized using UCSF Chimera and BIOVIA Discovery Studio, enabling direct inspection of residue positioning, local environment alterations, and potential disruption of key molecular interactions within the GAT1 architecture.

### 3.3. Binding Pocket Prediction Analysis

The spatial orientation of ligands within a protein’s holo-structure plays a critical role in accurately identifying the functional binding pocket of the target protein [31]. In line with recent findings demonstrating GAT1 inhibition by the drug tiagabine, the key binding-pocket residues were compiled based on previously published structural and functional studies. These residues were then used as the foundation for subsequent analyses in this work.

### 3.4. Molecular Docking Analysis

Molecular docking of tiagabine with all seven GAT1 mutant models was carried out using two complementary docking platforms, AutoDock 4.2 and Boltz-2, to ensure robust and cross-validated assessment of ligand–protein interactions. In AutoDock 4.2 [32], polar hydrogens and Kollman charges were assigned to the receptor, while Gasteiger charges were applied to tiagabine, with nonpolar hydrogens merged. All rotatable bonds in the ligand were kept flexible to allow full conformational freedom during docking. A grid box of 60 × 60 × 60 Å^3^ was centered on the GAT1 binding pocket, and 100 independent genetic algorithm (LGA) runs were performed using default empirical free-energy scoring parameters. The final docked conformations were ranked according to the lowest binding energy values (kcal/mol).

To complement the AutoDock evaluation, Boltz-2 (https://colab.research.google.com/github/kimjc95/computational-chemistry/blob/main/Boltz_on_Colab.ipynb) (accessed on 20 November 2025) scoring was also applied to each protein–ligand pair. This tool provides a physics-based binding affinity metric derived from thermodynamic principles, offering an additional layer of validation for the predicted docking trends. The combined use of AutoDock 4.2 and Boltz-2 ensured that both empirical docking energetics and statistical–thermodynamic affinity estimates were considered when assessing the impact of each mutation on tiagabine binding.

### 3.5. Residual Mutation Analysis

The chosen mutated structures were employed to forecast their impact on protein structures and related disorders. Specifically, seven mutations, G63S, Y140C, Q291Δ, F294Δ, N310I, D451G, and G457H, were selected based on information available in the literature [33]. Additionally, each mutated model underwent individual structural analysis using BIOVIA Discovery Studio [34] and the UCSF Chimera tool [35].

### 3.6. Molecular Dynamics (MD) Simulation

The seven mutated docked complexes, along with the wild-type GAT1 complexed with tigabine, underwent a 100 ns MD simulation. To assess the real-time residual flexibility of the predicted mutated structures in comparison to the wild type, the MD simulations were run in triplicate for each mutated model.

The CHARMM-GUI server’s solution builder protocol facilitated the generation of the CHARMM36 force field and preparation of input files for MD simulations in GROMACS. This involved several steps: (1) Uploading the GAT1 in complex with tiagabine to the CHARMM-GUI server. (2) Using the TIP3P solution to solvate the model in a periodic rectangular box extended 10Å beyond each peptide’s atom, employing KCl as the basic ion type and setting ion concentration to 0.15 with a Monte Carlo ion implementation method. (3) Adjusting the box dimensions to 94Å along each axis in the solvator step, resulting in a total system size of approximately 830,584 cubic Angstroms. The LINCS method constrained bonds, and the Verlet cutoff approach with a 10Å cutoff was used for electrostatic and Van der Waals interactions. The particle mesh Ewald (PME) method computed electrostatic interactions. (4) Subjecting the solvated systems to two equilibration phases under NVT and NPT conditions, with a simulation temperature of 30 °C. (5) Using a Python 3.11.1 format conversion tool provided by CHARMM-GUI to generate GROMACS topology (top) and parameter (itp) files for MD simulations on the Linux operating system with GROMACS (version 2019.3). The production dynamics ran in GROMACS with a 2fs time step, and coordinates for each picosecond were stored for subsequent MD analysis.

### 3.7. Free Energy Calculation

The gmx_MMPBSA v1.6.3 package was utilized to calculate end-state binding free energies for the protein–ligand systems using MD trajectories generated with GROMACS [36]. This analysis was performed using the MM/PBSA approach, which estimates binding energetics by separately evaluating the energies of the solvated protein–ligand complex, the unbound receptor, and the isolated ligand. The binding free energy (ΔG_binding) for all the triplicates was obtained according to the expression:

ΔG_binding_ = G_complex_ − (G_protein_ + G_ligand_) (1)

where *G_complex_* corresponds to the total free energy of the protein–ligand complex, and *G_protein_* and *G_ligand_* denote the energies of the individual solvated protein and ligand, respectively. This framework provides a quantitative measure of ligand affinity based on post-simulation energetic decomposition.

## 4. Conclusions

This comprehensive computational study provides a detailed mechanistic understanding of how seven clinically relevant GAT1 mutations, G63S, Y140C, Q291Δ, F294Δ, N310I, D451G, and G457H, alter the structural stability, conformational dynamics, and inhibitor-binding behavior of the human GAT1 transporter. Through an integrative analysis combining homology modeling, docking, molecular dynamics simulations, and free-energy profiling, we demonstrate that each mutation perturbs the GAT1 structure in distinct yet functionally significant ways. While the wild-type GAT1 displayed optimal compactness, low fluctuation, and the strongest binding affinity toward tiagabine, all mutant variants showed measurable deviations from this baseline, reflecting varying degrees of structural destabilization and compromised ligand accommodation.

The deletion mutants Q291Δ and F294Δ, along with the substitutions G457H, D451G, and N310I, produced the most pronounced destabilizing effects, including increased backbone fluctuations, elevated solvent exposure, disrupted hydrogen bonding, weaker interaction energies, and reduced binding free energies. These perturbations collectively indicate compromised inhibitor-binding efficiency and potential impairment of transporter function. In contrast, G63S and Y140C exhibited relatively moderate disruptions, retaining partial structural stability but still demonstrating weakened tiagabine interactions due to changes in local polarity and aromatic contacts.

Importantly, the consistency across RMSD, RMSF, R_g_, interaction energy, ΔG, and hydrogen-bond analyses strengthens the reliability of the observed trends and highlights the sensitivity of the GAT1 binding pocket to residue-specific modifications. The results provide clear mechanistic insight into how pathogenic mutations may alter GABA transport, inhibitor sensitivity, and therapeutic responsiveness. Collectively, this work not only advances our understanding of GAT1 structural biology but also establishes a robust computational foundation for guiding future experimental studies, rational drug design, and mutation-specific therapeutic strategies targeting dysfunctional GABAergic signaling.

## Figures and Tables

**Table 1:** The structural validation values of seven mutated models are assessed by online servers and compared with the wild-type GAT1.

Sr No	Mutated Structures	MolProbity Score	ProSA Z-Score	ERRAT Score	Ramachandran Favored
1	G63S	1.72	−5.12	98.05	97.3%
2	Y140C	1.59	−5.06	97.85	97.5%
3	Q291Δ	1.61	−4.99	97.85	97.3%
4	F294Δ	0.65	−5.07	98.83	97.9%
5	N310I	1.51	−5.15	98.43	97.5%
6	D451G	1.58	−5.06	97.86	97.5%
7	G457H	1.52	−5.18	98.63	97.3%
8	Wild Type	1.75	−5.15	91.27	97.0%

**Table 2:** The ∆∆G was calculated by different bioinformatics methods. Negative values indicate destabilization of the protein.

Mutations	PolyPhen2	MutPred2	ENCoM	DynaMut2 (kcal/mol)	DUET (kcal/mol)
G63S	1.00	0.906	0.188	−0.68	−1.466
Y140C	1.00	0.955	−0.971	−0.02	−1.055
Q291Δ	-	-	-	-	-
F294Δ	-	-	-	-	-
N310I	1.00	0.952	0.153	−0.59	−0.127
D451G	0.969	0.959	−0.457	1.38	1.928
G457H	1.00	0.944	0.941	−1.23	−2.107

**Table 3:** The calculated MMPBSA energy of the tiagabine bound to the mutated models.

Sr No	Mutation	R1	R2	R3	Average
		kcal/mol	kcal/mol	kcal/mol	
1	G63S	−27.88	−27.00	−25.69	−26.85
2	Y140S	−24.44	−26.07	−25.47	−25.32
3	Q291Δ	−23.87	−19.12	−22.04	−21.67
4	F294Δ	−27.02	−25.43	−24.64	−25.69
5	N310I	−22.65	−27.23	−20.82	−23.56
6	D451G	−18.72	−25.79	−25.04	−23.18
7	G457H	−23.99	−16.28	−25.56	−21.94
8	Wild	−31.07	−27.31	−26.48	−28.28

## Data Availability

The data supporting this study’s findings are available from the corresponding author upon reasonable request.

## Supplementary Materials

The following supporting information can be downloaded at: https://www.mdpi.com/article/10.3390/ijms262311339/s1.
