# Supplementary material for: Computational Analysis of GAT1 Mutations: Functional Consequences from Molecular Dynamics and Binding Free Energy Calculations
Source: Int J Mol Sci. 2025 Nov 24;26(23):11339. doi: 10.3390/ijms262311339 (PMC12692106; doi:10.3390/ijms262311339)
Supplement: Supplementary file 1 [file ijms-26-11339-s001.zip › ijms-3950777-supplementary.pdf]

Article

Computational Analysis of GAT1 Mutations: Functional Consequences from Molecular Dynamics and Binding Free Energy Calculations

Muhammad Yasir [1], Jinyoung Park [1], Eun-Taek Han [2], Won Sun Park [3], Jin-Hee Han [2], Jongseon Choe [4], Mubashir Hassan [5], Andrzej Kloczkowski [5,6,7] and Wanjoo Chun [1,*]

[1]Department of Pharmacology, Kangwon National University School of Medicine, Chuncheon, 24341, Republic of Korea;

[2]Department of Medical Environmental Biology and Tropical Medicine, Kangwon National University School of Medicine, Chuncheon, 24341, Republic of Korea;

[3]Department of Physiology, Kangwon National University School of Medicine, Chuncheon, 24341, Republic of Korea;

[4]Department of Microbiology and Immunology, Kangwon National University School of Medicine, Chuncheon, 24341, Republic of Korea

[5]The Steve and Cindy Rasmussen Institute for Genomic Medicine at Nationwide Children's Hospital, Columbus, OH 43205, USA

[6]Department of Pediatrics, The Ohio State University, Columbus, OH 43205, USA

[7]Department of Biomedical Informatics, The Ohio State University, Columbus, OH 43210, USA

*Correspondence: author: Dr. Wanjoo Chun, Department of Pharmacology Kangwon National University School of Medicine, Kangwon National University, Email: wchun@kangwon.ac.kr, Phone: +82-33-250-8853.

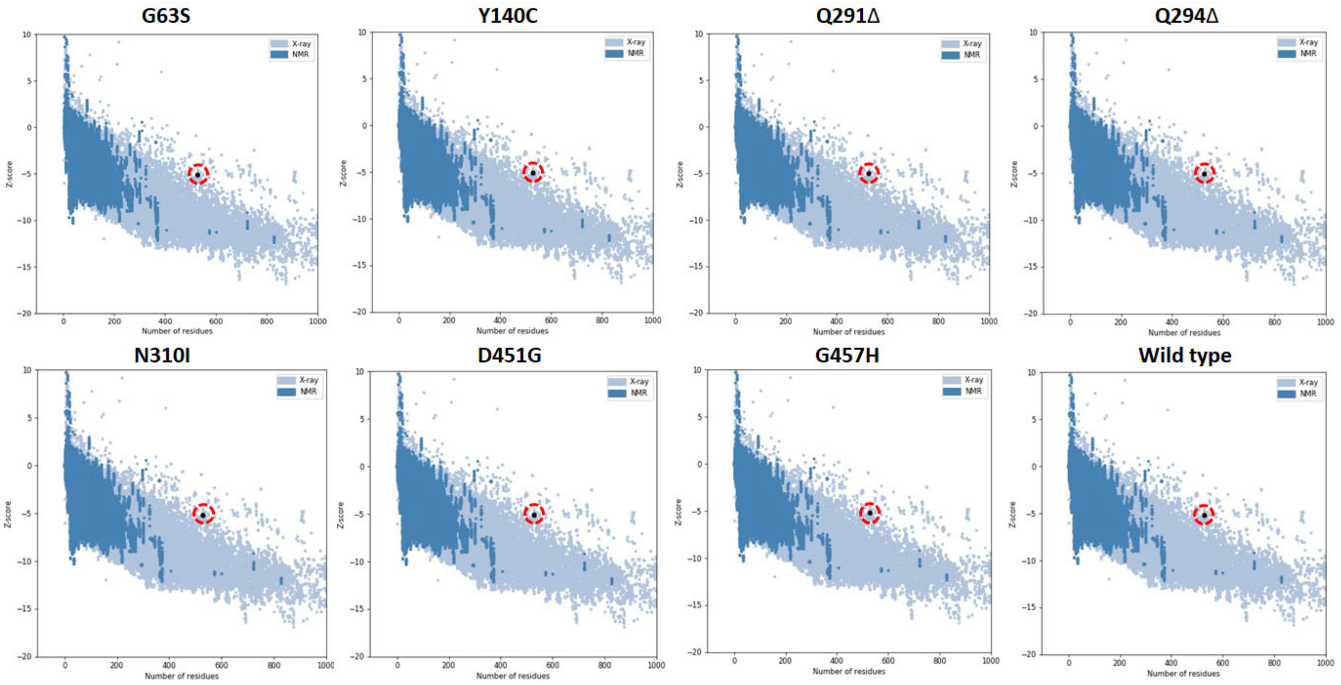

Figure S1. The ProSA results are visually represented in graphs, comparing the mutated models with X-ray and NMR structures retrieved from the Protein Data Bank (PDB).

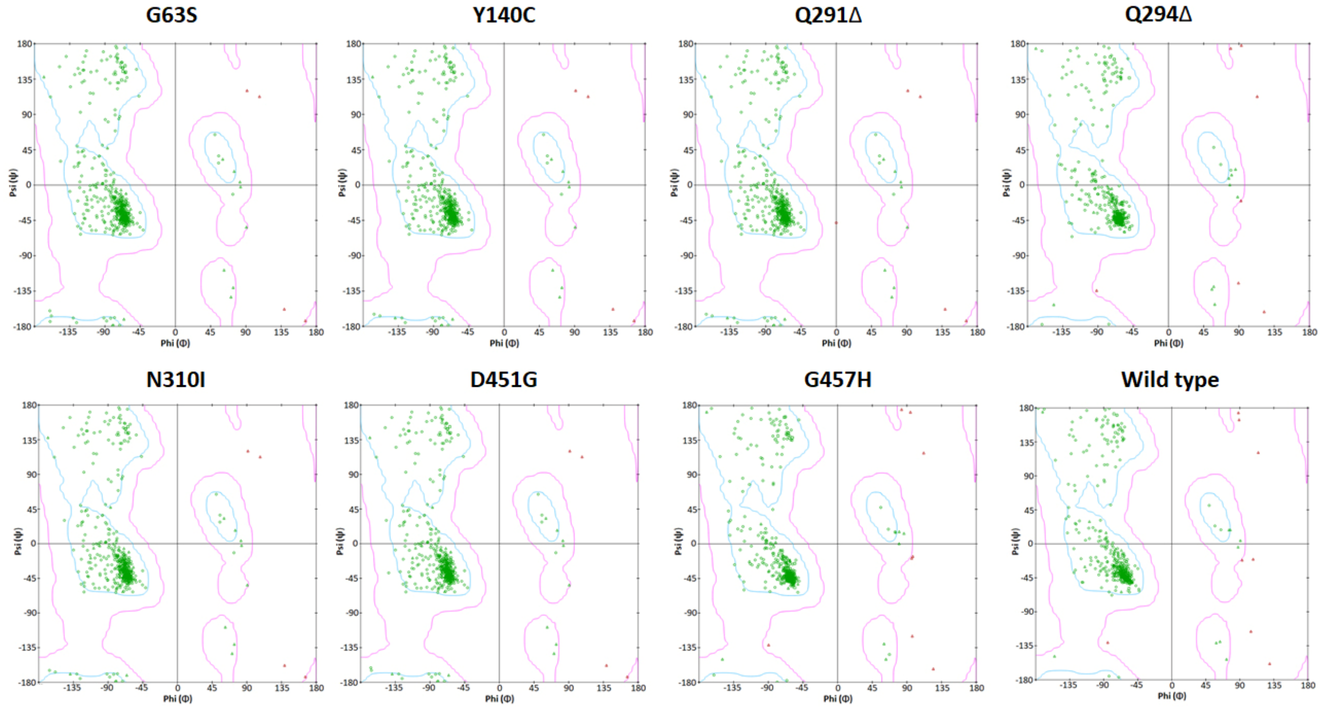

Figure S2. The Ramachandran graphs illustrate the conformational characteristics of all mutated models in comparison with the wild type. Amino acid residues are color-coded in green, with the favorable region marked by cyan boundaries and the disallowed region highlighted in pink.

Table S1. The molecular docking score of tiagabine to wild and mutated GAT1.

**Sr No**	**Mutation**	**Boltz-2 Binding Affinity**	**AutoDock**
1	G63S	-0.2586	-5.49
2	Y140S	-0.2792	-5.54
3	Q291Δ	-0.2568	-5.71
4	F294Δ	-0.3352	-5.62
5	N310I	0.2570	-6.85
6	D451G	-0.8051	-6.80
7	G457H	-0.5196	-5.20
8	Wild	-1.2298	-6.05

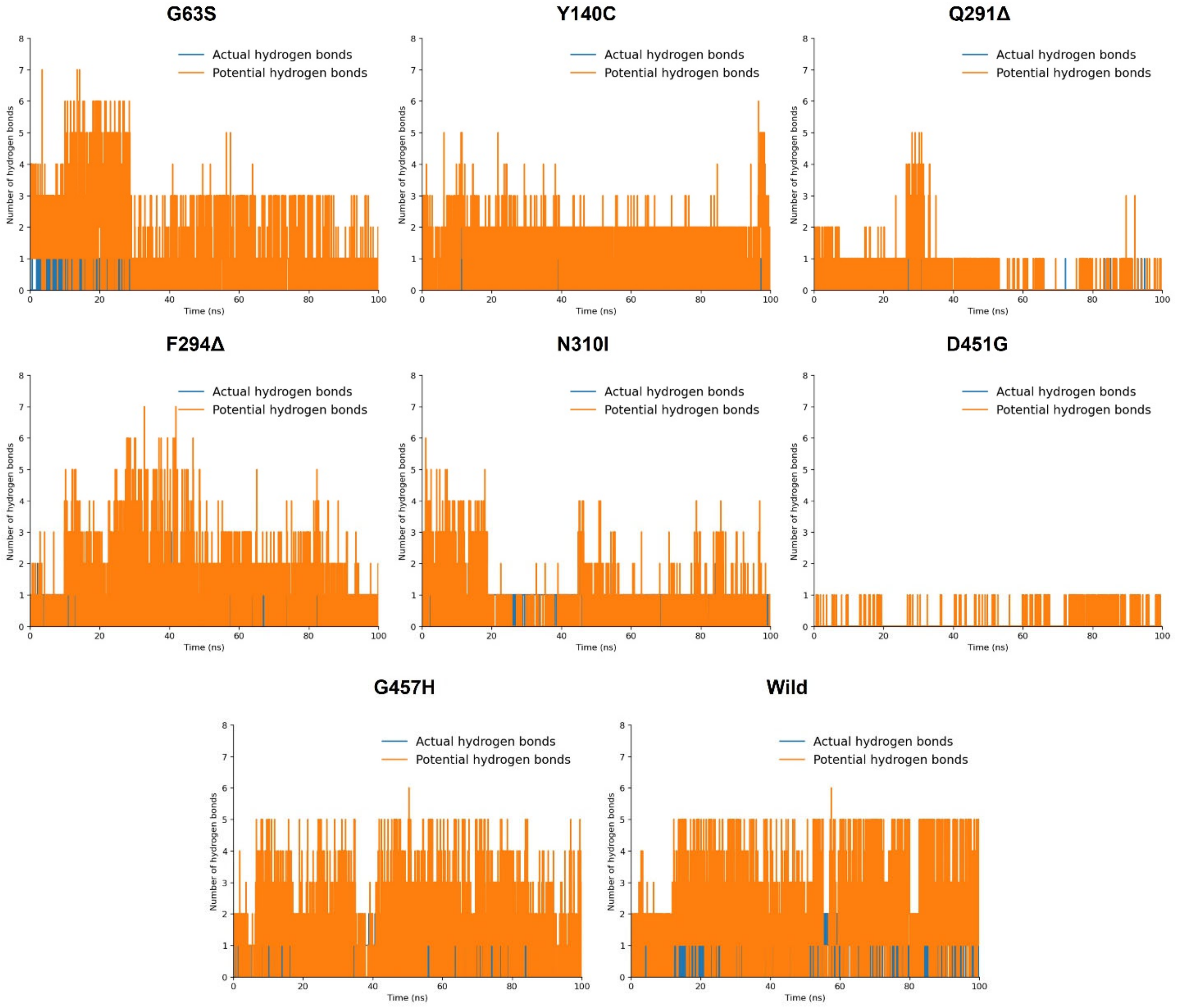

Figure S3. The hydrogen bond graphs of all mutated models in comparison with the wild type are depicted in this figure. Actual hydrogen bonds are manifested in blue color, while the potential hydrogen bonds within 0.35nm are manifested in orange color.
